# Muscle metabolism and activation heterogeneity by combined ^31^P chemical shift and T_2_ imaging, and pulmonary O_2_ uptake during incremental knee-extensor exercise

**DOI:** 10.1152/japplphysiol.00510.2013

**Published:** 2013-06-27

**Authors:** Daniel T. Cannon, Franklyn A. Howe, Brian J. Whipp, Susan A. Ward, Dominick J. McIntyre, Christophe Ladroue, John R. Griffiths, Graham J. Kemp, Harry B. Rossiter

**Affiliations:** ^1^Rehabilitation Clinical Trials Center, Division of Respiratory & Critical Care Physiology & Medicine, Los Angeles Biomedical Research Institute at Harbor-UCLA Medical Center, Torrance, California;; ^2^School of Biomedical Sciences, University of Leeds, Leeds, United Kingdom;; ^3^Department of Biochemistry, St George's, University of London, London, United Kingdom;; ^4^Department of Physiology, St George's, University of London, London, United Kingdom;; ^5^Division of Clinical Sciences, St George's, University of London, London, United Kingdom;; ^6^Human Bio-Energetics Research Centre, Crickhowell, Powys, United Kingdom;; ^7^Centre for Exercise Science and Medicine, University of Glasgow, Glasgow, United Kingdom;; ^8^Cancer Research UK Cambridge Research Institute, Li Ka Shing Centre, Cambridge, United Kingdom;; ^9^Department of Computer Science, University of Warwick, Coventry, United Kingdom; and; ^10^Department of Musculoskeletal Biology and Magnetic Resonance & Image Analysis Research Centre, University of Liverpool, Liverpool, United Kingdom

**Keywords:** skeletal muscle, exercise, quadriceps, magnetic resonance spectroscopy, oxygen uptake

## Abstract

The integration of skeletal muscle substrate depletion, metabolite accumulation, and fatigue during large muscle-mass exercise is not well understood. Measurement of intramuscular energy store degradation and metabolite accumulation is confounded by muscle heterogeneity. Therefore, to characterize regional metabolic distribution in the locomotor muscles, we combined ^31^P magnetic resonance spectroscopy, chemical shift imaging, and T_2_-weighted imaging with pulmonary oxygen uptake during bilateral knee-extension exercise to intolerance. Six men completed incremental tests for the following: *1*) unlocalized ^31^P magnetic resonance spectroscopy; and *2*) spatial determination of ^31^P metabolism and activation. The relationship of pulmonary oxygen uptake to whole quadriceps phosphocreatine concentration ([PCr]) was inversely linear, and three of four knee-extensor muscles showed activation as assessed by change in T_2_. The largest changes in [PCr], [inorganic phosphate] ([Pi]) and pH occurred in rectus femoris, but no voxel (72 cm^3^) showed complete PCr depletion at exercise cessation. The most metabolically active voxel reached 11 ± 9 mM [PCr] (resting, 29 ± 1 mM), 23 ± 11 mM [Pi] (resting, 7 ± 1 mM), and a pH of 6.64 ± 0.29 (resting, 7.08 ± 0.03). However, the distribution of ^31^P metabolites and pH varied widely between voxels, and the intervoxel coefficient of variation increased between rest (∼10%) and exercise intolerance (∼30–60%). Therefore, the limit of tolerance was attained with wide heterogeneity in substrate depletion and fatigue-related metabolite accumulation, with extreme metabolic perturbation isolated to only a small volume of active muscle (<5%). Regional intramuscular disturbances are thus likely an important requisite for exercise intolerance. How these signals integrate to limit muscle power production, while regional “recruitable muscle” energy stores are presumably still available, remains uncertain.

tolerance to whole body exercise is a major determinant of quality of life and mortality (42). However, the mechanisms limiting exercise tolerance are poorly understood. Skeletal muscle substrate depletion and other intramuscular metabolic changes contribute directly to limiting muscle power production (15). In addition, increased muscle energetic requirements may also contribute indirectly to exercise intolerance through a sense of effort, or by causing systemic metabolic disturbances that alter, for example, blood flow (Q̇) distribution, ventilation, the work of breathing, dyspnea, and/or pain (23). Reserves of whole muscle intramuscular energy stores [e.g., phosphocreatine (PCr) and adenosine triphosphate (ATP)] often remain at volitional exhaustion during large muscle mass exercise, such as bilateral knee/hip extension or cycle ergometry (22, 49). However, the magnitude of local variation in metabolic strain, and therefore whether the relevant muscles remain energetically competent to produce the required power during large muscle-mass exercise, remains obscure.

Human single muscle fibers sampled through biopsy manifest a wide distribution of metabolite concentrations during high-intensity exercise, with PCr concentration ([PCr]) in some fibers approaching the limit of detection even before the limit of tolerance (32). However, the extent to which these limiting conditions in a small biopsy reflect heterogeneous metabolic responses across the involved muscle group during exercise is not clear. While single-site metabolite estimates from biopsy (55) provide highly specific and localized information from the presumed most energetically challenged region of the muscle, measures such as muscle-venous blood sampling (which represents a flow-weighted mean) or unlocalized^1^ ^31^P magnetic resonance spectroscopy [(MRS) (52)] interrogate relatively large regions of heterogeneous muscle. During single-leg exercise, unlocalized ^31^P-MRS data suggest that exercise limitation coincides with a challenge to the muscle [PCr] as it approaches the limit of detection (58). It remains unclear, however, how much whole muscle, or even regional muscle, substrate depletion may occur during exercise with a larger involved muscle mass, where the systemic challenge to homeostasis and the influence of muscle afferent feedback is heightened. The combination of the heterogeneity in muscle metabolism and the technical limitations of available sampling methods greatly confound the in vivo characterization of skeletal muscle fatigue-related changes in relation to exercise limitation in large muscle-mass exercise. A “middle ground” of spatial resolution is offered by ^31^P chemical shift imaging (CSI), which may shed light on this complexity.

Detecting localized metabolic disturbances during exercise is possible with ^31^P-CSI (7). CSI can provide a metabolite map, allowing characterization of metabolism between muscles and within single-muscle regions. The method also can balance a need for spatial resolution against the short acquisition times necessary for the temporal resolution required in non-steady-state exercise. CSI has been used to study leg muscles at rest and in recovery from exercise (16, 27, 41), in forearm (21, 44) and dorsi-/plantar flexor (19, 48) muscles during steady-state exercise, and in the quadriceps muscles during constant-power single-leg exercise and recovery (45), although not during a dynamic bilateral leg activity. In addition, muscle metabolic heterogeneity at the limit of tolerance will reflect the diversity of muscle recruitment, as well as the biochemistry of supporting energy conversion. The tissue water spin-spin transverse relaxation time (T_2_) from ^1^H magnetic resonance imaging (MRI) provides the opportunity to estimate muscle activation patterns (1) in relation to measures of local (^31^P-CSI) and whole body [pulmonary oxygen uptake (V̇o_2_)] metabolism during bilateral leg exercise in the bore of a superconducting magnet (62).

Our aim was, therefore, to characterize the dynamic interrelationships between whole body V̇o_2_, whole and regional quadriceps ^31^P metabolism and intracellular pH (pH_i_), together with muscle activation by ^1^H-MRI, across the full range of exercise intensity. To achieve this, we used ^31^P-CSI of one thigh and T_2_-weighted ^1^H MRI of the other thigh during bilateral ramp-incremental knee extension to the limit of tolerance. We hypothesized that localized measurements would reveal potential regional metabolic limitation, which is obscured by traditional global measures of metabolism (V̇o_2_) and whole quadriceps ^31^P-MRS.

## MATERIALS AND METHODS

### 

#### Ethical approval.

The ethics committee at St. George's Hospital Trust, London, approved this study, and all procedures complied with the latest revision of the Declaration of Helsinki. Written, informed consent was obtained from all volunteers before their participation in the study.

#### Participants and exercise protocols.

Six healthy, active men were recruited for this study (mean ± SD: 24 ± 3 yr; 184 ± 3 cm; 88 ± 7.6 kg). Participants were screened for known disease and completed an exercise history and a physical activity readiness questionnaire.

All exercise tests were completed on an magnetic resonance (MR)-compatible, computer-controlled electromagnetically braked knee-extension ergometer (MRI Ergometer Up/Down, Lode BV, Groningen, the Netherlands). The participants lay prone with their feet strapped into molded plastic stirrups attached to aluminum arms linking the ergometer cranks. Their hips were secured to the scanner bed with padded nylon and Velcro straps to minimize hip flexion/extension. This setup, similar to an exercise model previously described (62), allowed for ∼35° (limited by the scanner bore) of bilateral, alternate knee-flexion/extension to drive an electromagnetically braked flywheel. No resistance was present during knee flexion, other than the work required to lift the mass of the lower leg.

Once familiarized, the physiological responses to this exercise mode are reproducible between visits in the same subjects (62). Therefore, each participant underwent a comprehensive series of familiarization visits to account for learning effects and also to ensure consistent muscle activation patterns among multiple MR measurements. Participants completed up to six ramp incremental tests until exercise responses could be reproduced on three successive occasions.

**Fig. 1.**
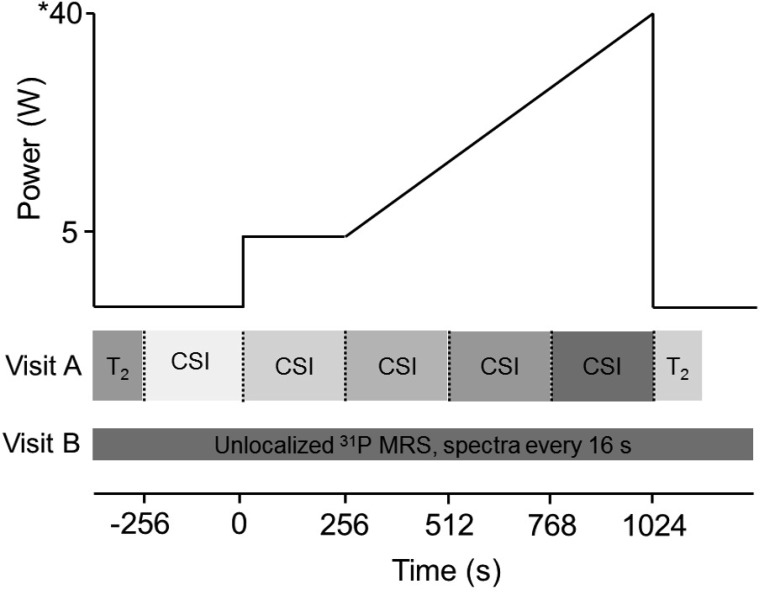
Composite schematic of the measurement protocols during two ramp incremental exercise tests. The protocol consisted of a resting stage, a 5-W baseline exercise stage, and a ramp incremental exercise phase tailored to bring about intolerance after 3 complete chemical shift imaging (CSI) stages. For CSI, the data-acquisition stages each lasted 256 s. On the *y*-axis, * denotes an example peak power, but this varied for each participant. T_2_, transverse relaxation time; MRS, magnetic resonance spectroscopy.

For the MR measurements, participants completed two ramp incremental exercise tests in a random order on separate days for *1*) CSI and T_2_ imaging; and *2*) unlocalized ^31^P-MRS. Participants lay at rest, followed by a baseline exercise phase at 5 W. The power was then increased continuously at a rate (2–3 W/min), tailored to each participant, to produce task failure in ∼12 min. This allowed for three complete CSI acquisitions during ramp incremental exercise (Fig. 1). The frequency of bilateral knee extension was 60 cycles/min, synchronized with ^31^P-MRS acquisition. Audible clicks within the scanner were achieved by including a pulsed spoiler gradient at the end of each MRS acquisition. Exercise was terminated when the participant could no longer maintain the required cadence, despite strong verbal encouragement. Participants lay still in the MR scanner for 5 min resting recovery, for ^31^P-MRS (following the unlocalized protocol) and thigh T_2_ image acquisition (following the CSI protocol). Pulmonary V̇o_2_ was simultaneously measured breath by breath (62).

#### ^31^P-MRS.

Muscle phosphorus-containing metabolites were measured with a 1.5T superconducting magnet (Signa Advantage, GE Healthcare, Milwaukee, WI). A one-pulse ^31^P-MRS acquisition was employed with an 8 in./5 in. transmit/receive surface coil placed under the knee extensors [rectus femoris (RF), vastus medialis (VM), vastus intermedius (VI), and vastus lateralis (VL)] of the dominant leg (the right leg in all subjects), halfway between hip and knee. A series of axial gradient-recalled echo images of the thigh confirmed coil placement relative to the knee-extensor muscles. Before the ^31^P acquisitions, the magnetic field homogeneity was optimized using the localized water signal from a large volume of quadriceps muscles enclosing the sensitive region of the ^31^P coil.

Unlocalized ^31^P free induction decays were collected from the quadriceps with a repetition time (TR) of 2 s throughout the rest-exercise-recovery protocol. ^31^P data were averaged over eight free induction decay acquisitions, yielding a spectrum every 16 s to estimate the signal intensities of Pi, PCr, γ-ATP, α-ATP, and β-ATP.

During the ramp incremental protocol, spatially resolved acquisition relied on ^31^P-CSI with 8-cm slice thickness, 16 × 16 phase encoding steps, and a 48-cm^2^ field of view. The volume of each voxel was 3 × 3 × 8 cm, or 72 cm^3^, and voxel selection for specific muscles was done with reference to the multislice MRI. A TR of 1 s was used, and a two-dimensional ^31^P metabolite map was generated every 256 s. This acquisition time was determined from pilot measurements to ensure good spectral data from the quadriceps and represents an optimal compromise between signal-to-noise, temporal resolution, and spatial resolution.

#### Data analyses for ^31^P measures.

Signal intensities, frequencies, and line widths of Pi, PCr, and ATP were quantified using Java-based version of the magnetic resonance user interface (43). Uncalibrated concentrations for PCr and Pi were determined from assuming β-ATP concentration of 8.2 mM (28, 54). pH_i_ was estimated from the chemical shift of Pi (40):

(1) pH=6.75+log(δ−3.27/5.69−δ)

where δ is the chemical shift of the Pi peak relative to PCr. In the case of Pi peak splitting (51, 57), a Pi signal intensity-weighted mean of the two pH values was used for analyses (53).

^31^P-CSI postprocessing was done using SAGEIDL software (GE, Milwaukee, WI, version dev2000.3) to allow for voxel shifting to the proper region of tissue to characterize the knee-extensor muscles. Signal intensities, frequencies, and line widths were transformed into a time series for each region of interest on the ^31^P metabolite map.

#### ^1^H-MRI.

At rest and immediately following the limit of tolerance, T_2_-weighted ^1^H images were obtained with the whole body imaging coil and used to estimate muscle activation. Changes in the spin-spin relaxation time, T_2_ (ΔT_2_) from rest to peak exercise were used to estimate relative muscle activation during ramp incremental exercise. The ^31^P surface coil under the right quadriceps caused artifact in the T_2_ image, and, therefore, ΔT_2_ was determined in the muscles of the left leg. Quantitative apparent-T_2_ maps were constructed pixel by pixel from dual-fast-spin echo ^1^H images (TR of 4,000 ms; echo times of 30 and 63 ms), assuming exponential decay, and quantified from the average of the density map within each quadriceps muscle. Scan time for T_2_ imaging was 176 s.

#### Pulmonary gas exchange.

Participants breathed through a low-resistance (<0.1 kPa·l^−1^·s^−1^ at 15 l/s), low-dead-space (90 ml) mouthpiece for the measurement of respired gases, as previously described (62). Flow rates and volumes were measured with a custom-designed nonmagnetic turbine flow sensor (VMM, Interface Associates, Laguna Niguel, CA), while a quadrupole mass spectrometer measured gas concentrations from respired gas sampled from the mouthpiece (QP9000; CaSE, Gillingham, Kent, UK). During the ^31^P-MRS experiment, the mass spectrometer and computer were housed in the scanner control room, outside the Faraday cage. The volume transducer signal was filtered to prevent conduction of radio frequency noise into the scanner room. Due to the length of the respired gas capillary tubing (13.7 m), the measured time delay between the near-instantaneous turbine flow signal and the gas concentration signals was used to align the signals for breath-by-breath analysis (6). This increase in transit delay and sample line length did not influence the 5–95% rise time of the quadrupole mass spectrometer or the signal fidelity of respired gases (62).

The flow sensor and gas analyzers were calibrated before each experiment. The turbine volume transducer was calibrated in the bore of the scanner with a 3-liter syringe (Hans Rudolph, Shawnee, KS). The calibration was completed with flow rates ranging from 0.2 to 6 l/s, mimicking flow rates expected for humans at rest and during exercise. After the completion of the flow sensor calibration, the flow volumes were verified over 25 syringe strokes of varying flow rates and accepted when the means were within ±0.01 l, with a SD and coefficient of variation (CV) of 0.02 l and 1%, respectively. Additionally, the mass spectrometer was calibrated with two high-precision-certified gases (BOC Group, Guildford, UK) with concentrations of O_2_, CO_2_, and N_2_ spanning the physiological range (O_2_, 21 and 10%; CO_2_, 0 and 8%; N_2_, 79 and 82%), and verified using room air. Following each experiment, the mass spectrometer calibrations were checked by resampling the calibration gases.

#### Data analyses for pulmonary gas exchange.

To improve signal to noise, V̇o_2_ breath-by-breath data were filtered by removing values residing more than 4 SD from the local mean. Data were time aligned, interpolated, averaged into 10-s bins, and ensemble averaged across like transitions. Peak values were calculated from 20-s means immediately before the limit of tolerance.

#### Statistical analyses.

Changes in V̇o_2_, muscle activity, and metabolites with exercise were assessed with repeated-measures ANOVA or paired *t*-test, where appropriate. Heterogeneity was characterized using a normalized dispersion, or CV (expressed as %). Statistics were completed using the Statistical Package for the Social Sciences (SPSS v17.0, SPSS, Chicago, IL).

## RESULTS

**Fig. 2.**
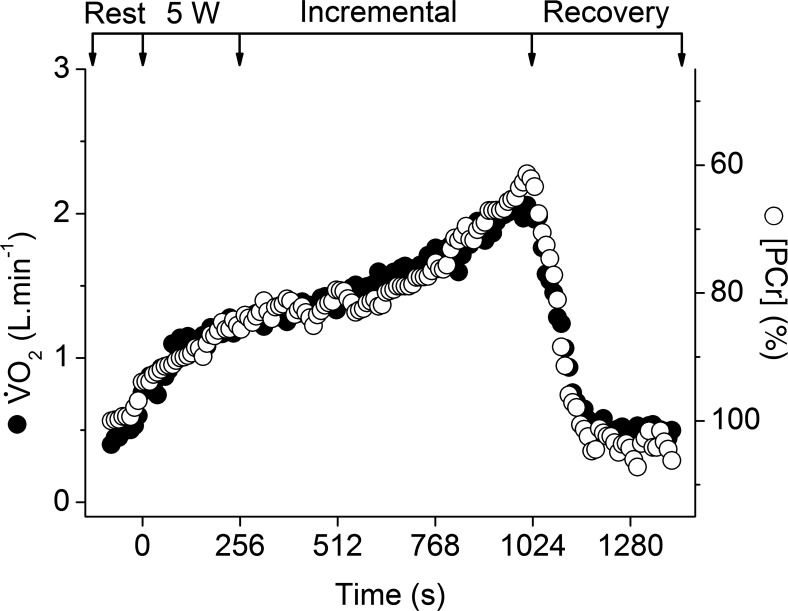
Profile of O_2_ uptake (V̇o_2_; ●) and whole-quadriceps unlocalized phosphocreatine concentration ([PCr]; ○) during the ramp incremental exercise phase in a representative participant. Arrows represent the beginning/end of 5-W and ramp incremental phases. [PCr] is given as percentage of resting, with the *y*-axis inverted to show more clearly the relationship to V̇o_2_.

**Fig. 3.**
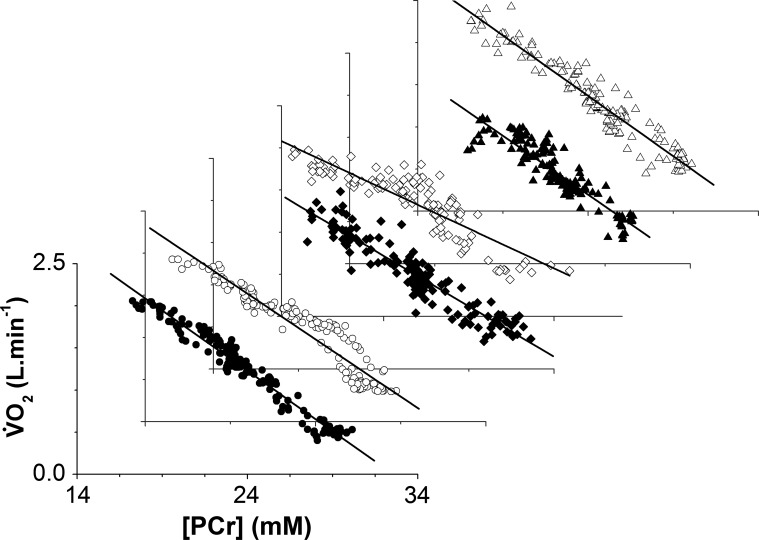
Profiles of the V̇o_2_-[PCr] relationship during the ramp incremental exercise and recovery in 6 participants. Resting and recovery data are included. [PCr] is given as an absolute concentration. A linear regression line is shown for each individual data set. For clarity, the symbols represent different participants.

During ramp incremental exercise, participants achieved a peak power of 42 ± 10 W, which was reproducible within 2 ± 2 W. Following an initial kinetic phase, V̇o_2_ rose progressively to achieve a peak V̇o_2_ of 1.98 ± 0.30 l/min, which equated to ∼55% of cycling maximum V̇o_2_ in these participants. Consequently, the dynamic V̇o_2_-[PCr] relationship from the unlocalized ^31^P-MRS measurements was approximately linear in all participants (Figs. 2 and 3). The mean slope and intercept of this relationship were −0.13 ± 0.02 l·min^−1^·mM^−1^ and 4.34 ± 0.53 l/min, respectively. The CV for the slope and intercept of the V̇o_2_-[PCr] were ∼15% for each of the participants, with the regression function for five of the participants showing a slope and intercept CV of ∼7%.

Resting [PCr], [Pi], and pH_i_ from unlocalized MRS were 29 ± 1 mM, 7 ± 1 mM, and 7.08 ± 0.03, respectively. At peak exercise, [PCr], [Pi], and pH_i_ were 20 ± 4 mM, 19 ± 5 mM, and 6.91 ± 0.08. Therefore, from rest to peak exercise, [PCr] decreased by 9 ± 4 mM (*P* = 0.002), [Pi] increased by 12 ± 4 mM (*P* = 0.001), and pH_i_ decreased (*P* = 0.001) by 0.18 ± 0.07.

T_2_ imaging revealed activation variability in the four quadriceps muscles contributing to the exercise. In the RF muscle, T_2_ increased from rest to exercise (47 ± 1.6 vs. 58 ± 8.2 ms; *P* = 0.02), while no change was detected in the VM muscle (*P* = 0.15), VI (*P* = 0.22), or VL (*P* = 0.22) (Table 1). However, this approach obscured individual differences in T_2_ changes among muscles, as there was a considerable variability in activation pattern of the knee extensors among participants (24). Therefore, the postexercise muscle activation was ranked from largest to smallest T_2_ change for each participant. A significant main effect was present in ranked muscle activation by ΔT_2_ (*F* = 5.9; *P* = 0.007; η^2^ = 0.54), with two of four rank orderings different from one another (Table 2), indicating large variability in activation among muscles. Additionally, three of four muscles showed a ΔT_2_ that was different from zero, indicating that at least three of the four knee-extensor muscles were active (to varying degrees) in each participant (Table 2). At the limit of tolerance, T_2_ heterogeneity in each participant was −21% to +32% of the knee-extensor mean T_2_.

**Table 1.** T_2_ at rest and immediately after the limit of tolerance in the muscles of the quadriceps

	RF		VM		VI		VL	
Participant No.	Preexercise	Postexercise	Preexercise	Postexercise	Preexercise	Postexercise	Preexercise	Postexercise
*1*	45	49	52	51	63	64	63	52
*2*	48	63	51	55	59	62	52	75
*3*	46	62	49	52	63	66	54	56
*4*	46	70	51	57	61	60	56	63
*5*	49	55	46	49	47	57	35	51
*6*	47	50	48	45	49	49	49	51
Mean (SD)	47 (2)	58 (8)*	49 (2)	52 (4)	57 (7)	60 (6)	51 (9)	58 (10)

Values are in ms. T_2_, spin-spin relaxation time; RF, rectus femoris; VM, vastus medialis; VI, vastus intermedius; VL, vastus lateralis. Different from the preexercise value (*P = 0.02).

**Table 2.** ΔT_2_ in the muscles of the quadriceps

	Largest ΔT2		2nd Largest ΔT2		2nd Smallest ΔT2		Smallest ΔT2	
Participant No.	ΔT2	Muscle	ΔT2	Muscle	ΔT2	Muscle	ΔT2	Muscle
*1*	4	RF	1	VI	−1	VM	−11	VL
*2*	23	VL	15	RF	4	VM	3	VI
*3*	16	RF	3	VM	3	VI	2	VL
*4*	24	RF	7	VL	6	VM	−1	VI
*5*	16	VL	10	VI	6	RF	3	VM
*6*	3	RF	2	VL	0	VI	−3	VM
Mean (SD)	14 (9)†		6 (5)*†		3 (3)‡		−1 (5)§	

Difference in T_2_ relaxation times between rest and immediately after the limit of tolerance (ΔT_2_). Values are presented in ms and ordered from largest to smallest ΔT_2_. Different from the next largest rank-ordering of ΔT_2_ (

* *P* = 0.02,

§ *P* = 0.03). Different from zero (

† *P* = 0.05,

‡ *P* = 0.06).

**Fig. 4.**
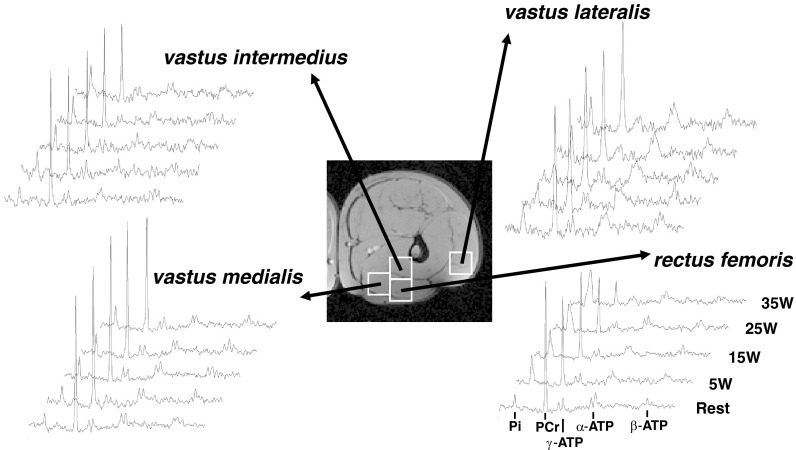
^31^P-MRS stack plots acquired by CSI in a representative participant during each stage of the exercise protocol from 4 voxels placed in each of the 4 knee-extensor muscles labeled. ^31^P-MR spectra, from *bottom* to *top*, represent rest, 5-W baseline exercise, and three acquisitions during ramp incremental exercise. Visible in the spectra are the peaks (chemical shift from *left* to *right*) for Pi, PCr, γ-ATP, α-ATP, and β-ATP. The two-dimensional ^1^H magnetic resonance image was used to identify voxel positioning.

**Fig. 5.**
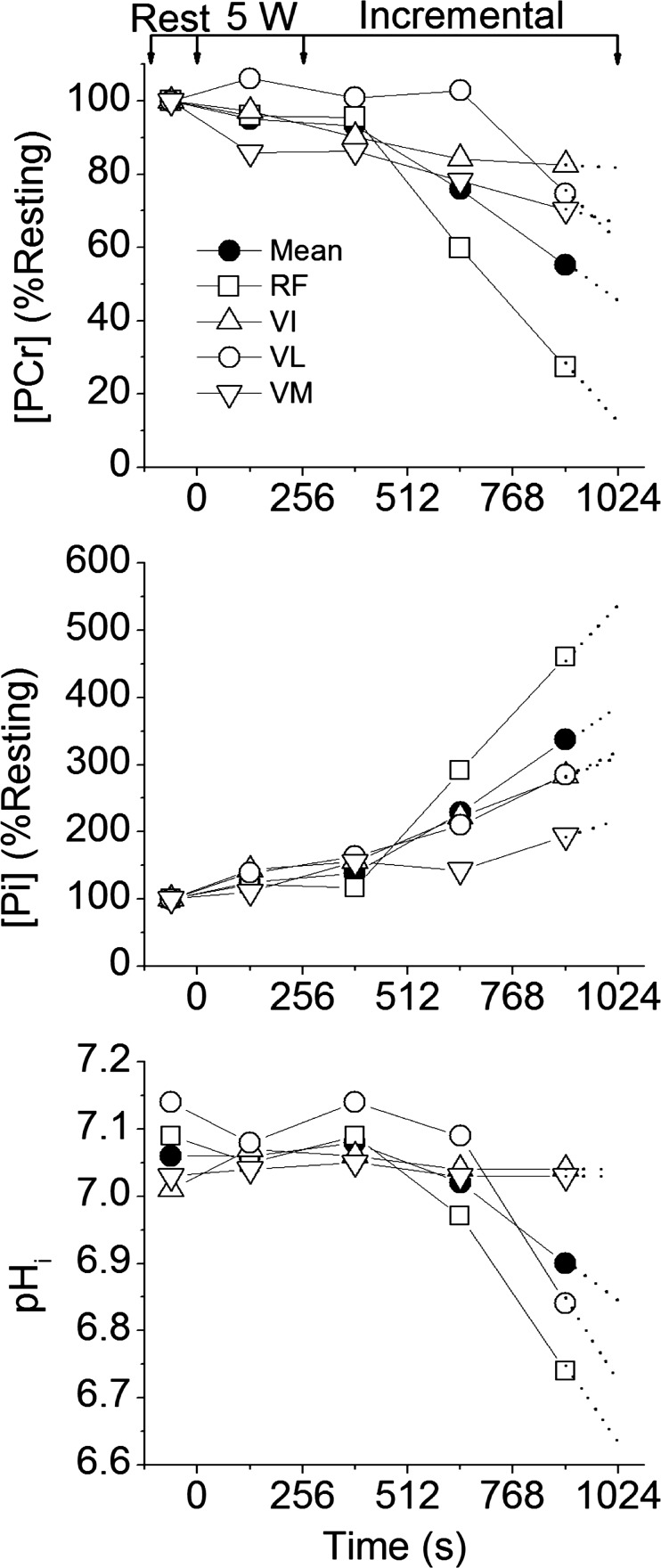
Profiles for [PCr], [Pi], and intracellular pH (pH_i_) measured by CSI in a representative participant in the 4 knee-extensor muscles: rectus femoris (RF; □); vastus intermedius (VI; △); vastus lateralis (VL; ○); vastus medialis (VM; ▽). Also shown is the mean of the 4 muscles (●). Arrows represent the beginning/end of 5-W and ramp incremental phases. [PCr] and [Pi] are expressed as a percentage of their resting values. Dotted lines show linear regression extrapolation to the limit of tolerance.

**Fig. 6.**
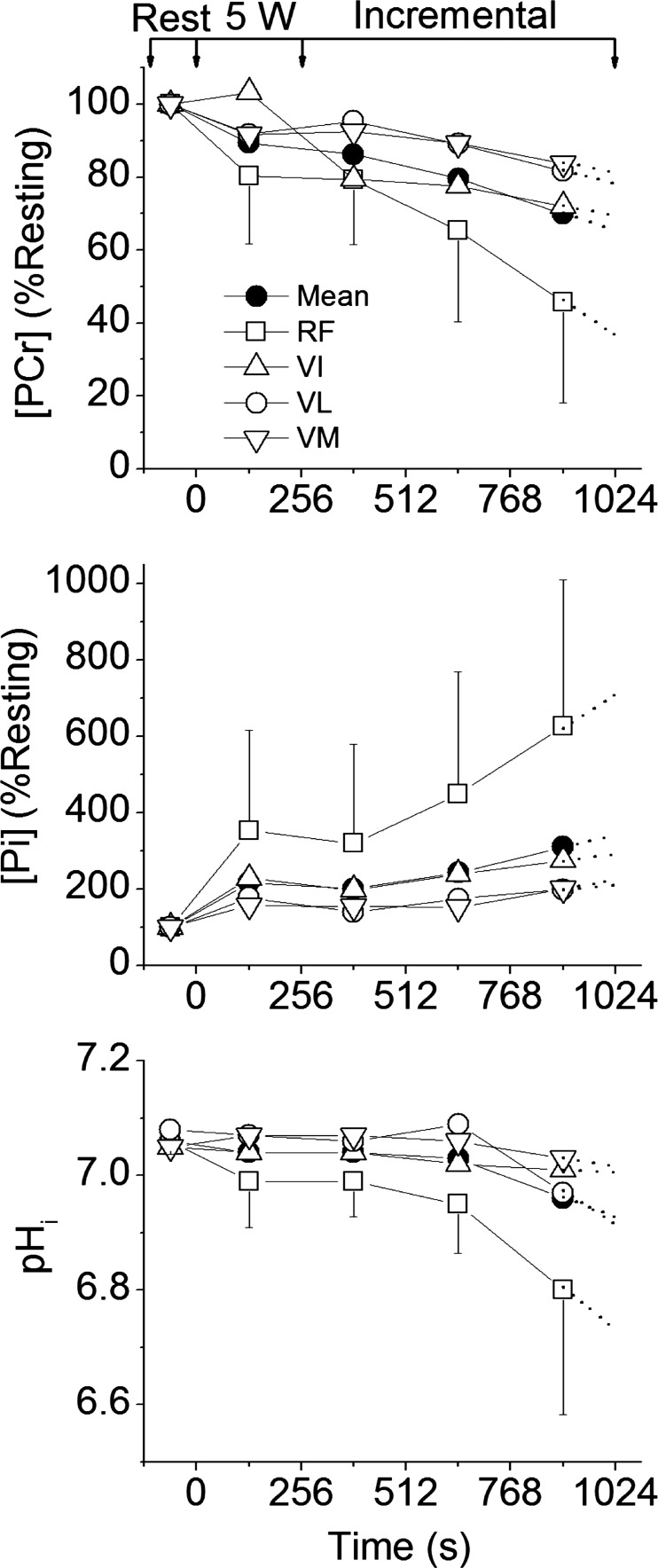
Group means for [PCr], [Pi], and pH_i_ measured by CSI in the 4 knee-extensor muscles: RF, VI, VL, VM. Also shown is the mean of the 4 muscles. Symbols are as defined in Fig. 5 legend. Arrows represent the beginning/end of 5-W and ramp incremental phases. Error bars for the VI, VL, and VM are omitted for clarity, but show similar distribution to the RF. Dotted lines show linear regression extrapolation to the limit of tolerance.

**Fig. 7.**
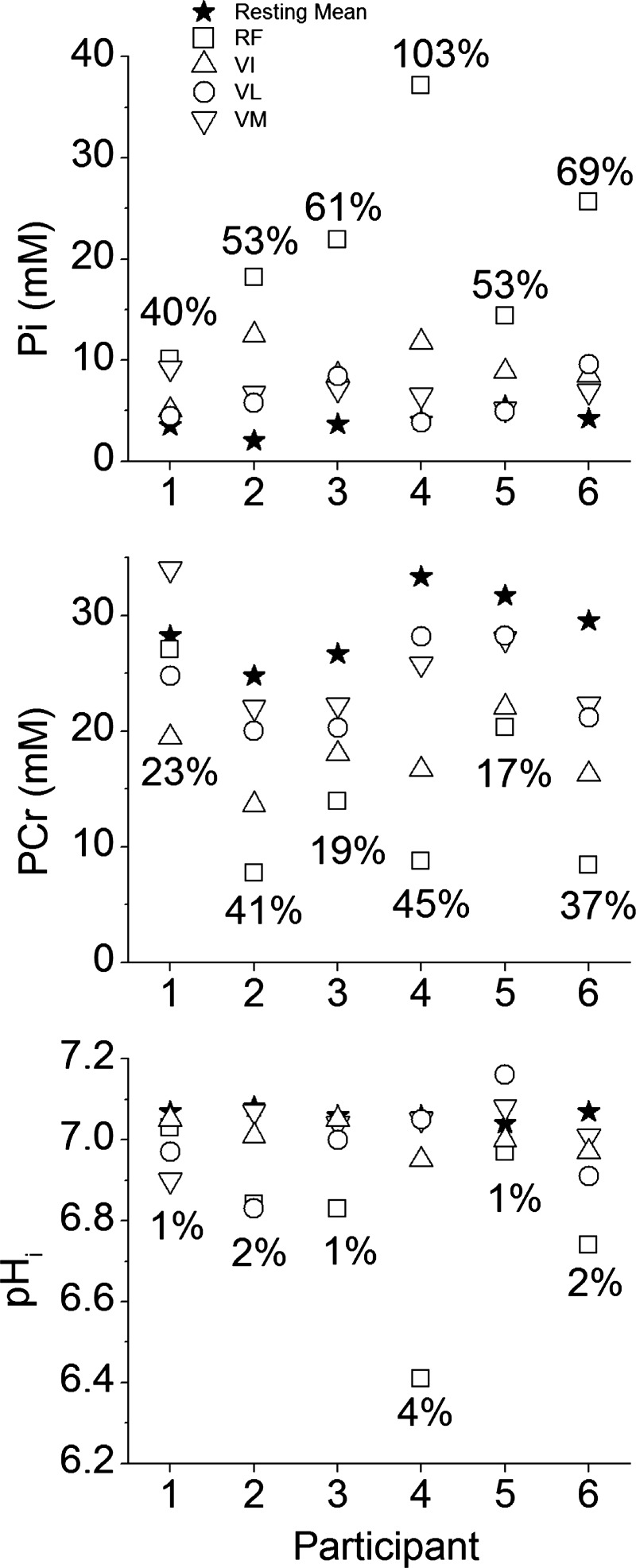
Individual participant data for [PCr], [Pi], and pH_i_ measured by CSI at peak exercise in the 4 knee-extensor muscles: RF, VI, VL, VM. Symbols are as defined in Fig. 5 legend. The resting value (★) is the mean of all 4 knee extensors. Coefficients of variation are presented as a percentage on each panel and represent the value at peak exercise.

[PCr], [Pi], and pH_i_ changes from rest to end-exercise varied considerably across the knee-extensor muscles (Figs. 4, 5, and 6). The largest changes in each variable occurred in the RF muscle, with a significant interaction (power × muscle) present (*F* = 10.0; *P* = 0.02; η^2^ = 0.67). No single voxel showed “complete” depletion of PCr; the lowest concentration (in RF) reaching ∼8 mM in three participants (Fig. 7). The CV for ^31^P metabolites at each power across the knee-extensor muscles increased from rest to peak exercise ([PCr], 11 ± 5 vs. 30 ± 12%, *P* = 0.01; [Pi], 35 ± 9 vs. 63 ± 22%, *P* = 0.03). The relatively small CV for pH_i_ (∼1–4%) reflects its logarithmic scale; thus, for [H^+^], CV increased from rest to peak exercise (6 ± 3 vs. 34 ± 26%; *P* = 0.04). As the CSI metabolite maps are a mean of the entire ∼4-min acquisition, the trajectories of metabolite changes were extrapolated to exercise cessation using linear regression between the final two CSI acquisitions in the ramp incremental protocol (Figs. 5 and 6). At the point of intolerance [PCr], [Pi], and pH_i_ in the RF were extrapolated to 11 ± 9 mM (or 36 ± 27% resting), 23 ± 11 mM, and 6.64 ± 0.29, respectively. Extrapolated [PCr] in two participants was reduced to 3.5 mM, or ∼10% of resting [PCr] (e.g., Fig. 5).

At peak exercise, Pi peak splitting was evident in the RF voxel of all six participants. Additionally, all participants showed at least three of four knee extensors with Pi peak splitting during exercise. When pHi was calculated from the chemical shift of Pi, the difference at peak exercise for the split Pi voxels in the RF muscle was 6.70 ± 0.23 vs. 6.92 ± 0.18 (*P* = 0.0006).

The changes in [PCr], [Pi], and pH_i_ from rest to end exercise were used to rank the four muscles of the quadriceps in order of metabolic disturbance, as described above for T_2_. A significant effect was present for ranked Δ[PCr] (*F* = 13.8; *P* = 0.01; η^2^ = 0.74), Δ[Pi] (*F* = 19.5; *P* = 0.004; η^2^ = 0.80), and ΔpH_i_ (*F* = 9.9; *P* = 0.02; η^2^ = 0.66) (Tables 3, 4, and 5). This analysis indicated that the metabolic disturbance in the four muscles (reduction in [PCr], and increase in [Pi]) was significantly different from one another (Tables 3 and 4). This was also the case for ΔpH_i_, except that the two muscles showing the greatest change (mostly RF and VL) were not different from each other (Table 5).

**Table 3.** Δ[PCr] in the muscles of the quadriceps

	Largest Δ[PCr]		2nd Largest Δ[PCr]		2nd Smallest Δ[PCr]		Smallest Δ[PCr]	
Participant No.	Δ[PCr]	Muscle	Δ[PCr]	Muscle	Δ[PCr]	Muscle	Δ[PCr]	Muscle
*1*	9.6	VI	6.5	RF	3.5	VM	2.2	VL
*2*	15.1	RF	7.9	VI	6.7	VL	5.4	VM
*3*	16.5	RF	8.9	VI	5.3	VM	3.5	VL
*4*	27.2	RF	10.3	VI	2.9	VM	−4.0	VL
*5*	10.6	RF	9.9	VI	5.7	VL	3.2	VM
*6*	20.3	RF	13.7	VI	10.3	VM	5.7	VL
Mean (SD)	16.5 (6.5)		9.5 (2.5)*		5.7 (2.7)†		2.7 (3.6)‡	

Difference in phosphocreatine concentration between rest and end-exercise (Δ[PCr]) values are presented in mM and ordered from largest to smallest Δ[PCr]. Different from the next largest rank-ordering of Δ[PCr] (

* *P* = 0.03,

† *P* = 0.01,

‡ *P* = 0.02).

**Table 4.** Δ[Pi] in the muscles of the quadriceps

	Largest Δ[Pi]		2nd Largest Δ[Pi]		2nd Smallest Δ[Pi]		Smallest Δ[Pi]	
Participant No.	Δ[Pi]	Muscle	Δ[Pi]	Muscle	Δ[Pi]	Muscle	Δ[Pi]	Muscle
*1*	7.3	RF	6.1	VM	2.1	VI	−0.3	VL
*2*	16.5	RF	8.8	VI	4.9	VM	4.5	VL
*3*	19.7	RF	4.6	VL	4.1	VI	3.4	VM
*4*	30.3	RF	8.5	VI	3.0	VM	0.5	VL
*5*	10.1	RF	3.6	VI	2.1	VM	−2.5	VL
*6*	20.1	RF	6.2	VL	5.5	VI	3.3	VM
Mean (SD)	17.3 (8.2)		6.3 (2.1)*		3.6 (1.4)†		1.5 (2.7)†	

Difference in Pi concentration between rest and end-exercise (Δ[Pi]) values are presented in mM and ordered from largest to smallest Δ[Pi]. Different from the next largest rank-ordering of Δ[Pi] (

* *P* = 0.01,

† *P* = 0.02).

**Table 5.** ΔpH_i_ in the muscles of the quadriceps

	Largest ΔpH_i_		2nd Largest ΔpH_i_		2nd Smallest ΔpH_i_		Smallest ΔpH_i_	
Participant No.	ΔpH_i_	Muscle	ΔpH_i_	Muscle	ΔpH_i_	Muscle	ΔpH_i_	Muscle
*1*	0.14	VM	0.12	VL	0.06	VI	0.02	RF
*2*	0.22	RF	0.19	VL	0.05	VI	−0.04	VM
*3*	0.24	RF	0.06	VL	0.01	VM	−0.01	VI
*4*	0.61	RF	0.06	VL	0.06	VI	0.02	VM
*5*	0.10	RF	0.08	VI	−0.01	VM	−0.04	VL
*6*	0.34	RF	0.19	VL	0.07	VM	0.06	VI
Mean (SD)	0.28 (0.18)		0.12 (0.06)		0.04 (0.03)*		0.002 (0.04)†	

Difference in intramuscular pH between rest and end-exercise (ΔpH_i_) values are ordered from largest to smallest ΔpH_i_. Different from the next largest rank-ordering of ΔpH_i_ (

* *P* = 0.01,

† *P* = 0.02).

## DISCUSSION

### 

#### Activation of the knee extensors and regional metabolism.

The lagged profiles of whole body V̇o_2_ and unlocalized [PCr] were consistent with an approximately exponential response to the linear power forcing (Fig. 2). The resulting slopes of the linear phase of the dynamic V̇o_2_-[PCr] relationship were similar among participants, reflecting that the whole-muscle oxidative capacity, total creatine concentration, and ATP rephosphorylated per ½ O_2_ reduced (P/O) varied only slightly among these young healthy individuals (37) (Fig. 3). However, consistent with our hypothesis, this linearity obscured a wide distribution of ^31^P metabolite changes across the knee-extensor muscles during ramp incremental exercise, which was revealed by localized ^31^P-CSI (Figs. 6 and 7). The heterogeneity of [PCr] and [Pi] between individual muscles approximately tripled from rest to the limit of tolerance, and the heterogeneity of [H^+^] increased approximately fivefold.

This “metabolic strain” is related to muscle activation and the metabolic properties of the muscle region. Muscle activation was assessed in the present study by the change in T_2_ (38). ΔT_2_ reflects mainly the accumulation of osmotically active ions and water associated with muscle activation (1). During our knee-extensor exercise protocol, over the range of extension from ∼145 to 180°, the greatest disturbance to [PCr], [Pi], and pH_i_ (Figs. 5 and 6) and ΔT_2_ (Table 2) was most commonly seen in the RF muscle. However, muscle activation patterns were highly variable among individuals for this exercise mode, prompting comparisons using the rank order of muscle activity. Using this approach, at the limit of tolerance, three of four knee-extensor muscles showed at least some activation (ΔT_2_ different from zero; Table 2), consistent with being biomechanically “available” to contribute to power production during prone knee extension. Importantly, and contrary to our hypothesis, no single muscle region showed complete depletion of [PCr], even when responses were extrapolated to end-exercise from the time of the CSI acquisitions. Thus, our principal finding is that the limit of tolerance was reached with an apparent energy reserve of ∼10% of [PCr] in the most metabolically active muscle region, with heterogeneous metabolic responses across the active muscle group.

#### Considerations for spatial resolution.

The interacting constraints on spatial and temporal resolution for CSI are a potential limitation of our study. The voxel sizes (72 cm^3^) equate to ∼80 g of tissue (assuming a muscle tissue density of ∼1.1 g/cm^3^), rendering CSI a “blunt” tool compared with biopsy. The single-voxel mass (∼5% of the active muscle) greatly exceeds that of muscle biopsy (∼0.005% of active muscle) where single fibers have been reported with near-complete PCr depletion (31). To this point, numerous voxels in the present study exhibited Pi peak splitting, consistent with previous measurements (57), suggesting that intravoxel compartmentalization of metabolites was present in our data. Nevertheless, our data show that only very small, localized muscle regions are likely to demonstrate metabolically limiting conditions at the limit of tolerance during bilateral leg exercise. It also provides an important additional frame of reference for the distribution of the metabolic disturbance within the muscle mass available for exercise. This is because the data suggest that the true proportion of muscle mass reaching metabolically limiting conditions resides in between the resolutions of the CSI used here and the muscle biopsy technique.

From available biopsy data, ∼10% of muscle fibers in the sample exhibit [PCr] approaching zero (operationally, <5 mmol/kg dry mass) when sampled after submaximal heavy exercise (31); these fibers were part of a distribution with a mean [PCr] of ∼30 mmol/kg dry mass with a CV of ∼15% (31). Our data show a mean extrapolated [PCr] in the RF muscle at the limit of tolerance of ∼10 mM (wet mass), with a CV of ∼30%. Therefore, given the values from biopsy studies, it seems reasonable to suggest that [PCr] will approach zero in at least 10% of the muscle fibers within a highly metabolically active voxel. However, not all voxels characterizing the active muscle showed this magnitude of metabolic challenge. Across the entire mass engaged in exercise, the true regions with intracellular conditions limiting force production may be far smaller than local biopsy results suggest, and probably of the order of <5%, dispersed widely through the muscle i.e., PCr is depleted in ∼10% of the fibers within only one of the four active quadriceps muscles measured by CSI. In other words, intramuscular metabolic conditions that would severely impair muscle power production during voluntary large muscle-mass exercise are observed only in a very limited fraction of the recruited muscle. Whether these conditions, in very small muscle volumes, contribute to bringing about task failure remains unclear.

#### Heterogeneity in skeletal muscle perfusion and metabolism during exercise.

Within a limb muscle region during exercise, or even within a single muscle (13), Q̇ is unevenly distributed, which has the potential to disrupt oxygen transport and mitochondrial oxygen utilization (47). During isometric knee-extension in humans, the quadriceps muscle exhibits a wide heterogeneity of Q̇ (25). Our data also show wide variation in metabolic changes, with the RF muscle exhibiting the largest disturbance. Whether these two dispersions are well matched, or even related, remains uncertain.

The matching of Q̇ to V̇o_2_ is an important determinant of exercise tolerance (46, 61), so there is considerable interest in measuring both variables with high temporal and spatial resolution. Due to the technical constraints in achieving the required resolutions, this has proven to be a difficult task, even while at rest (39). Unlike in the myocardium (35) or brain (34), there appears to be little or no relationship between local Q̇ and metabolism (i.e., as judged by glucose uptake or PCr breakdown) in exercising skeletal muscle (20, 26, 36, 48). This is in contrast to measures made during rest and exercise-recovery in which Q̇-V̇o_2_ matching was relatively homogenous (39), or in the case of estimating metabolic activity from free fatty acid uptake, which was correlated with Q̇ (36). Measuring spatially resolved Q̇ with arterial spin labeling (ASL) in concert with PCr measurement in humans (48) showed a poor relationship of perfusion to metabolism, although differences in oxidative capacity between muscle regions complicate interpretation of these comparisons. Also, volume changes, movement artifact, and fluid redistribution each present a substantial technical challenge for ASL in contracting skeletal muscle, as opposed to its application to measuring brain Q̇ where these complications are mostly absent (56). Recent reports do show sufficient signal-to-noise with ASL in exercise recovery, where movement artifact and muscle volume change are smaller and slower compared with those during rhythmic contraction (64, 65). However, the estimation of local Q̇/V̇o_2_ during exercise through ASL and CSI has yet to be revisited since the initial methodological report (48). From the limited data available, it appears as though local Q̇ is poorly matched to local metabolism during exercise.

Additional evidence for the regional disruption of the balance between muscle O_2_ delivery and consumption is available using multichannel near-infrared spectroscopy. Near-infrared spectroscopy also indicates a poor relationship between local Q̇/V̇o_2_ with wide distributions in deoxygenation among quadriceps muscle regions during constant-power exercise (deoxygenation amplitude CVs ∼10–50%) (29). This is similar to the variation in metabolism for the large locomotor knee-extensor muscles during dynamic exercise observed in our study. However, without simultaneous high-resolution measurements of perfusion, the dynamic Q̇-V̇o_2_ relationships in skeletal muscle remain elusive.

#### Potential mechanisms for exercise limitation.

The last measurement in the CSI series represents the regional averages over the final ∼4 min of the ^31^P dynamics during ramp incremental exercise (Fig. 1). Therefore, the final CSI metabolite map best represents metabolite concentrations at ∼2 min before the point of intolerance. When the trajectories of metabolite changes are extrapolated to exercise cessation, assuming linear dynamics (60), an energy reserve still remains within even the most metabolically active muscle (Fig. 6, 11 ± 9 mM, or 36 ± 27% resting, 23 ± 11 mM, and 6.64 ± 0.29 for [PCr], [Pi], and pH_i_, respectively).

The presence of a remaining energy “reserve” at the limit of tolerance raises the question of what causes the exercise to be terminated. This finding is consistent with previous demonstrations and suggests that peripheral muscle “energetic failure” may not be responsible for exercise limitation (22, 49, 58). However, the regions of tissue that manifest the greatest disturbances in pH_i_, [Pi], and [PCr] may also be responsible for the generation of neural feedback signals that contribute to a sense of effort and therefore exercise limitation (4). Additionally, while the reduction in [PCr] fails to explain interference with power production per se (55), it is indicative of increases in [ADP], [Pi], and [H^+^] that cause muscle fatigue during high-intensity exercise through disruptions in cross-bridge cycling and Ca^2+^ handling (2). High [ADP] can disrupt power output (9), and Pi is inversely correlated with force output (63), likely due to the disruption in myosin detachment by competitive binding of ADP and Pi to the myosin head (15, 66). Further effects on power output with high [Pi] are expected from a reduction in Ca^2+^ sensitivity at troponin C and Ca^2+^ availability by precipitate formation in the sarcoplasmic reticulum (2, 59).

**Fig. 8.**
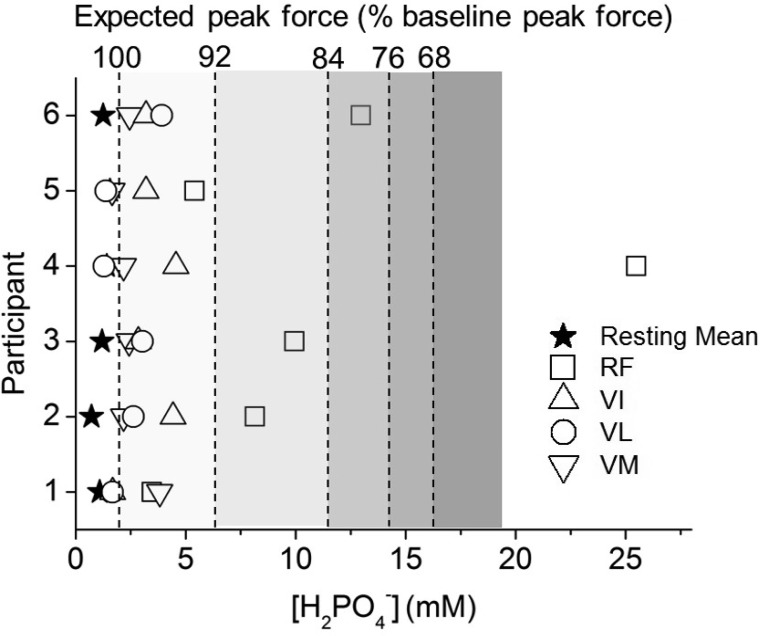
Calculated concentration of diprotonated (monanionic) Pi (H_2_PO_4_^−^) (63) plotted for each participant at rest and at the limit of tolerance of incremental exercise in the 4 knee-extensor muscles: RF, VI, VL, VM. Symbols are as defined in Fig. 5 legend. The resting value (★) is the mean of all 4 knee extensors. Shaded panels show expected peak force as a percentage of the baseline muscle value, calculated as a function of H_2_PO_4_^−^ concentration on the basis of the relationship observed in a study of human first dorsal interosseous muscle (8).

While the proposed role of pH_i_ in mammalian skeletal muscle fatigue has been questioned (59), some reports show that it may be responsible for fatigue at, or near, physiological temperatures via alterations in Ca^2+^ sensitivity (11, 15, 50). Additionally, changes in [Pi] and pH_i_ both contribute to increases in the diprotonated Pi species (H_2_PO_4_^−^) (63), which is implicated in fatigue. Whether or not the relationship between [H_2_PO_4_^−^] and decline in force production is causal, there is a strong correlation between muscle fatigue and [H_2_PO_4_^−^] (8), which can, therefore, be used as an index of the potential disruption in force production. Following this logic, the distribution of [H_2_PO_4_^−^] in the present data shows muscle regions that are expected to show a substantial reduction in peak force output at the limit of tolerance (Fig. 8) (8). This analysis suggests that peak force production in whole voxels in four of the six participants would be reduced by >10%. Whether this influence contributes to exercise limitation directly though failure of power production, or indirectly via feedback from muscle afferents, or some other process, remains to be determined. Nevertheless, while single voxels may demonstrate evidence of energetic fatigue in the present study, it appears that these regions reflect only a small proportion (<5%) of the active muscle mass.

It is currently unclear whether neural afferent feedback from stimuli arising in these regions is necessary for limiting exercise tolerance in healthy subjects. Studies using intrathecal fentanyl demonstrate that afferent feedback is vital for proper “calibration” of central motor output in healthy individuals (3–5), and that by blocking these signals it is possible to improve exercise tolerance, at least in chronic obstructive pulmonary disease (17). While the improvement in chronic obstructive pulmonary disease patient exercise tolerance was more likely from reduced ventilatory drive and reduced dyspnea, the signals generated in the periphery were presumably a driving force for a complex feedback cascade contributing to exercise limitation.

#### Implications for sampling techniques.

Metabolic distributions within skeletal muscle are largely attributed to the differing enzymatic and energetic profiles of type I and II muscle fibers, and the distribution of metabolites and substrate depletion in these fibers have traditionally been investigated through biopsy. Naturally, this technique provides the advantage of direct measurement of specific cellular metabolites. Biopsy studies suggest large reductions in PCr during high-intensity cycling exercise (and accumulation of Pi, ADP, and H^+^) (18, 31). However, these data are of limited use in determining the responses over an entire muscle or muscle group. Biopsy volume (∼50–300 mg) is typically assumed to be representative of the entire volume of muscle contributing to the exercise task [∼1–3 kg per leg, based on thigh mass of ∼5–7 kg in adult men (12)]. It is clear from the large variability in phosphorus metabolite concentrations reported here and elsewhere that the biopsy technique may well miss fibers that approach limiting levels or even show depletion of PCr. The biopsy technique also offers no spatial and temporal information, unless multiple sampling sites per muscle are used (raising obvious ethical and practical concerns).

Data in the literature and from our study therefore indicate that single-site sampling techniques, such as biopsy, are insufficient to characterize whole-muscle metabolism during voluntary exercise (24, 33). Based on the heterogeneity in skeletal muscle reported here and elsewhere (10, 14, 19, 24, 48), a greater spatial and temporal fidelity is needed to assess the changes in metabolic activity and fatigue-related metabolites in relation to limitation during large muscle-mass exercise.

#### Conclusions.

While whole body V̇o_2_ was linearly related to whole quadriceps [PCr] during ramp incremental exercise, the limit of tolerance was attained with a remaining intramuscular energy store and heterogeneous dynamics in all ^31^P variables examined. The CSI technique suggests that <5% of the quadriceps muscle volume was metabolically challenged at the limit of bilateral knee extension, despite activity in the majority of the quadriceps (based on T_2_ change) indicating the ability of these muscles to contribute power production. These data suggest that single-site sampling techniques are inadequate to characterize the heterogeneous nature of muscle metabolism during dynamic exercise in humans. While we have used a spatially resolved technique, which substantially improves interpretation for muscle metabolite changes over unlocalized ^31^P-MRS, Pi peak splitting was apparent in most muscle regions, indicating that further compartmentalization may mask regions of limitation. How these signals are integrated to bring about exercise intolerance in large muscle-mass exercise remains to be determined.

## GRANTS

This study was supported by Wellcome Trust, UK (058420 and 064898), and Biotechnology and Biological Sciences Research Council, UK (BB/I00162X/1 and BB/I001174/1).

## DISCLOSURES

No conflicts of interest, financial or otherwise, are declared by the author(s).

## AUTHOR CONTRIBUTIONS

Author contributions: D.T.C., F.A.H., C.L., and H.B.R. analyzed data; D.T.C., F.A.H., J.R.G., and H.B.R. interpreted results of experiments; D.T.C. prepared figures; D.T.C. and H.B.R. drafted manuscript; D.T.C., F.A.H., S.A.W., D.J.M., C.L., J.R.G., G.J.K., and H.B.R. edited and revised manuscript; D.T.C., F.A.H., S.A.W., D.J.M., C.L., J.R.G., G.J.K., and H.B.R. approved final version of manuscript; F.A.H., B.J.W., S.A.W., G.J.K., and H.B.R. conception and design of research; F.A.H., B.J.W., D.J.M., and H.B.R. performed experiments.

## Footnotes

1 While ^31^P-MRS is, by definition, surface-coil localized, we will refer to it as “unlocalized” throughout the paper.

## References

[1] Adams GR Duvoisin MR Dudley GA Magnetic resonance imaging and electromyography as indexes of muscle function. J Appl Physiol 73: 1578–1583, 1992 1447107 10.1152/jappl.1992.73.4.1578

[2] Allen DG Lamb GD Westerblad H Skeletal muscle fatigue: cellular mechanisms. Physiol Rev 88: 287–332, 2008 18195089 10.1152/physrev.00015.2007

[3] Amann M Blain GM Proctor LT Sebranek JJ Pegelow DF Dempsey JA Implications of group III and IV muscle afferents for high-intensity endurance exercise performance in humans. J Physiol 589: 5299–5309, 2011 21878520 10.1113/jphysiol.2011.213769 PMC3225681

[4] Amann M Dempsey JA Locomotor muscle fatigue modifies central motor drive in healthy humans and imposes a limitation to exercise performance. J Physiol 586: 161–173, 2008 17962334 10.1113/jphysiol.2007.141838 PMC2375542

[5] Amann M Proctor LT Sebranek JJ Pegelow DF Dempsey JA Opioid-mediated muscle afferents inhibit central motor drive and limit peripheral muscle fatigue development in humans. J Physiol 587: 271–283, 2009 19015193 10.1113/jphysiol.2008.163303 PMC2670040

[6] Beaver WL Wasserman K Whipp BJ On-line computer analysis and breath-by-breath graphical display of exercise function tests. J Appl Physiol 34: 128–132, 1973 4697371 10.1152/jappl.1973.34.1.128

[7] Bottomley PA Charles HC Roemer PB Flamig D Engeseth H Edelstein WA Mueller OM Human in vivo phosphate metabolite imaging with ^31^P NMR. Magn Reson Med 7: 319–336, 1988 3205148 10.1002/mrm.1910070309

[8] Cady EB Jones DA Lynn J Newham DJ Changes in force and intracellular metabolites during fatigue of human skeletal muscle. J Physiol 418: 311–325, 1989 2621621 10.1113/jphysiol.1989.sp017842 PMC1189973

[9] Cooke R Pate E The effects of ADP and phosphate on the contraction of muscle fibers. Biophys J 48: 789–798, 1985 3878160 10.1016/S0006-3495(85)83837-6 PMC1329404

[10] Damon BM Wadington MC Lansdown DA Hornberger JL Spatial heterogeneity in the muscle functional MRI signal intensity time course: effect of exercise intensity. Magn Reson Imaging 26: 1114–1121, 2008 18508220 10.1016/j.mri.2008.01.023 PMC2614829

[11] De Ruiter CJ De Haan A Temperature effect on the force/velocity relationship of the fresh and fatigued human adductor pollicis muscle. Pflügers Arch 440: 163–170, 2000 10864011 10.1007/s004240000284

[12] Dempster WT Space requirements of the seated operator: geometrical, kinematic, and mechanical aspects of the body, with special reference to the limbs. Wright-Patterson Air Force Base, OH: Wright Air Development Center US Air Force, 1955, p. 55–159

[13] Duling BR Damon DH An examination of the measurement of flow heterogeneity in striated muscle. Circ Res 60: 1–13, 1987 3552283 10.1161/01.res.60.1.1

[14] Endo MY Kobayakawa M Kinugasa R Kuno S Akima H Rossiter HB Miura A Fukuba Y Thigh muscle activation distribution and pulmonary V̇o_2_ kinetics during moderate, heavy, and very heavy intensity cycling exercise in humans. Am J Physiol Regul Integr Comp Physiol 293: R812–R820, 2007 17459915 10.1152/ajpregu.00028.2007

[15] Fitts RH The cross-bridge cycle and skeletal muscle fatigue. J Appl Physiol 104: 551–558, 2008 18162480 10.1152/japplphysiol.01200.2007

[16] Forbes SC Slade JM Francis RM Meyer RA Comparison of oxidative capacity among leg muscles in humans using gated ^31^P 2-D chemical shift imaging. NMR Biomed 22: 1063–1071, 2009 19579230 10.1002/nbm.1413

[17] Gagnon P Bussieres JS Ribeiro F Gagnon SL Saey D Gagne N Provencher S Maltais F Influences of spinal anesthesia on exercise tolerance in patients with chronic obstructive pulmonary disease. Am J Respir Crit Care Med 186: 606–615, 2012 22822019 10.1164/rccm.201203-0404OC

[18] Hargreaves M McKenna MJ Jenkins DG Warmington SA Li JL Snow RJ Febbraio MA Muscle metabolites and performance during high-intensity, intermittent exercise. J Appl Physiol 84: 1687–1691, 1998 9572818 10.1152/jappl.1998.84.5.1687

[19] Houtman CJ Heerschap A Zwarts MJ Stegeman DF pH heterogeneity in tibial anterior muscle during isometric activity studied by ^31^P-NMR spectroscopy. J Appl Physiol 91: 191–200, 2001 11408430 10.1152/jappl.2001.91.1.191

[20] Iversen PO Nicolaysen G Local blood flow and glucose uptake within resting and exercising rabbit skeletal muscle. Am J Physiol Heart Circ Physiol 260: H1795–H1801, 1991 10.1152/ajpheart.1991.260.6.H1795 2058716

[21] Jeneson JA Nelson SJ Vigneron DB Taylor JS Murphy-Boesch J Brown TR Two-dimensional ^31^P-chemical shift imaging of intramuscular heterogeneity in exercising human forearm muscle. Am J Physiol Cell Physiol 263: C357–C364, 1992 10.1152/ajpcell.1992.263.2.C357 1514584

[22] Jeneson JA Schmitz JP Hilbers PA Nicolay K An MR-compatible bicycle ergometer for in-magnet whole-body human exercise testing. Magn Reson Med 63: 257–261, 2010 19918886 10.1002/mrm.22179

[23] Jones NL Killian KJ Exercise limitation in health and disease. N Engl J Med 343: 632–641, 2000 10965011 10.1056/NEJM200008313430907

[24] Kalliokoski KK Boushel R Langberg H Scheede-Bergdahl C Ryberg AK Dossing S Kjaer A Kjaer M Differential glucose uptake in quadriceps and other leg muscles during one-legged dynamic submaximal knee-extension exercise. Front Physiol 2: 75, 2011 22046164 10.3389/fphys.2011.00075 PMC3200561

[25] Kalliokoski KK Kemppainen J Larmola K Takala TO Peltoniemi P Oksanen A Ruotsalainen U Cobelli C Knuuti J Nuutila P Muscle blood flow and flow heterogeneity during exercise studied with positron emission tomography in humans. Eur J Appl Physiol 83: 395–401, 2000 11138581 10.1007/s004210000267

[26] Kalliokoski KK Scheede-Bergdahl C Kjaer M Boushel R Muscle perfusion and metabolic heterogeneity: insights from noninvasive imaging techniques. Exerc Sport Sci Rev 34: 164–170, 2006 17031254 10.1249/01.jes.0000240018.07502.48

[27] Kan HE Klomp DW Wong CS Boer VO Webb AG Luijten PR Jeneson JA In vivo ^31^P MRS detection of an alkaline inorganic phosphate pool with short T1 in human resting skeletal muscle. NMR Biomed 23: 995–1000, 2010 20878975 10.1002/nbm.1517 PMC3856567

[28] Kemp GJ Meyerspeer M Moser E Absolute quantification of phosphorus metabolite concentrations in human muscle in vivo by 31P MRS: a quantitative review. NMR Biomed 20: 555–565, 2007 17628042 10.1002/nbm.1192

[29] Koga S Poole DC Fukuoka Y Ferreira LF Kondo N Ohmae E Barstow TJ Methodological validation of the dynamic heterogeneity of muscle deoxygenation within the quadriceps during cycle exercise. Am J Physiol Regul Integr Comp Physiol 301: R534–R541, 2011 21632845 10.1152/ajpregu.00101.2011

[31] Krustrup P Soderlund K Mohr M Bangsbo J The slow component of oxygen uptake during intense, sub-maximal exercise in man is associated with additional fibre recruitment. Pflügers Arch 447: 855–866, 2004 14758477 10.1007/s00424-003-1203-z

[32] Krustrup P Soderlund K Mohr M Gonzalez-Alonso J Bangsbo J Recruitment of fibre types and quadriceps muscle portions during repeated, intense knee-extensor exercise in humans. Pflügers Arch 449: 56–65, 2004 15290298 10.1007/s00424-004-1304-3

[33] Krustrup P Soderlund K Relu MU Ferguson RA Bangsbo J Heterogeneous recruitment of quadriceps muscle portions and fibre types during moderate intensity knee-extensor exercise: effect of thigh occlusion. Scand J Med Sci Sports 19: 576–584, 2009 18627560 10.1111/j.1600-0838.2008.00801.x

[34] Kuschinsky W Suda S Sokoloff L Local cerebral glucose utilization and blood flow during metabolic acidosis. Am J Physiol Heart Circ Physiol 241: H772–H777, 1981 10.1152/ajpheart.1981.241.5.H772 7304767

[35] L'Abbate A Camici P Trivella MG Pelosi G Regional myocardial glucose utilization assessed by (14C) deoxyglucose. Basic Res Cardiol 76: 394–398, 1981 7283943 10.1007/BF01908330

[36] Laaksonen MS Kemppainen J Kyrolainen H Knuuti J Nuutila P Kalliokoski KK Regional differences in blood flow, glucose uptake and fatty acid uptake within quadriceps femoris muscle during dynamic knee-extension exercise. Eur J Appl Physiol 113: 1775–1782, 2013 23417482 10.1007/s00421-013-2609-8

[37] Meyer RA Linear dependence of muscle phosphocreatine kinetics on total creatine content. Am J Physiol Cell Physiol 257: C1149–C1157, 1989 10.1152/ajpcell.1989.257.6.C1149 2610252

[38] Meyer RA Prior BM Functional magnetic resonance imaging of muscle. Exerc Sport Sci Rev 28: 89–92, 2000 10902092

[39] Mizuno M Kimura Y Iwakawa T Oda K Ishii K Ishiwata K Nakamura Y Muraoka I Regional differences in blood flow and oxygen consumption in resting muscle and their relationship during recovery from exhaustive exercise. J Appl Physiol 95: 2204–2210, 2003 12871962 10.1152/japplphysiol.00197.2003

[40] Moon RB Richards JH Determination of intracellular pH by 31P magnetic resonance. J Biol Chem 248: 7276–7278, 1973 4743524

[41] Morikawa S Inubushi T Kito K Tabata R Imaging of phosphoenergetic state and intracellular pH in human calf muscles after exercise by 31P NMR spectroscopy. Magn Reson Imaging 12: 1121–1126, 1994 7997099 10.1016/0730-725x(94)91244-q

[42] Myers J Prakash M Froelicher V Do D Partington S Atwood JE Exercise capacity and mortality among men referred for exercise testing. N Engl J Med 346: 793–801, 2002 11893790 10.1056/NEJMoa011858

[43] Naressi A Couturier C Devos JM Janssen M Mangeat C de Beer R Graveron-Demilly D Java-based graphical user interface for the MRUI quantitation package. MAGMA 12: 141–152, 2001 11390270 10.1007/BF02668096

[44] Nelson SJ Taylor JS Vigneron DB Murphy-Boesch J Brown TR Metabolite images of the human arm: changes in spatial and temporal distribution of high energy phosphates during exercise. NMR Biomed 4: 268–273, 1991 1816805 10.1002/nbm.1940040604

[45] Pesta D Paschke V Hoppel F Kobel C Kremser C Esterhammer R Burtscher M Kemp GJ Schocke M Different metabolic responses during incremental exercise assessed by localized 31P MRS in sprint and endurance athletes and untrained individuals. Int J Sports Med. In press 10.1055/s-0032-1327648 23378173

[46] Piiper J Perfusion, diffusion and their heterogeneities limiting blood-tissue O_2_ transfer in muscle. Acta Physiol Scand 168: 603–607, 2000 10759596 10.1046/j.1365-201x.2000.00711.x

[47] Piiper J Haab P Oxygen supply and uptake in tissue models with unequal distribution of blood flow and shunt. Respir Physiol 84: 261–271, 1991 1876763 10.1016/0034-5687(91)90122-y

[48] Richardson RS Haseler LJ Nygren AT Bluml S Frank LR Local perfusion and metabolic demand during exercise: a noninvasive MRI method of assessment. J Appl Physiol 91: 1845–1853, 2001 11568171 10.1152/jappl.2001.91.4.1845

[49] Rodenburg JB de Boer RW Jeneson JA van Echteld CJ Bar PR 31P-MRS and simultaneous quantification of dynamic human quadriceps exercise in a whole body MR scanner. J Appl Physiol 77: 1021–1029, 1994 8002487 10.1152/jappl.1994.77.2.1021

[50] Roots H Ball G Talbot-Ponsonby J King M McBeath K Ranatunga KW Muscle fatigue examined at different temperatures in experiments on intact mammalian (rat) muscle fibers. J Appl Physiol 106: 378–384, 2009 19057001 10.1152/japplphysiol.90883.2008 PMC2644245

[51] Rossiter HB Howe FA Ward SA Intramuscular phosphate and pulmonary V̇o_2_ kinetics during exercise. In: Oxygen Uptake Kinetics in Sport, Exercise, and Medicine, edited by Jones AM Poole DC London: Routledge, 2005, p. 154–184

[52] Rossiter HB Ward SA Doyle VL Howe FA Griffiths JR Whipp BJ Inferences from pulmonary O_2_ uptake with respect to intramuscular [phosphocreatine] kinetics during moderate exercise in humans. J Physiol 518: 921–932, 1999 10421675 10.1111/j.1469-7793.1999.0921p.x PMC2269465

[53] Rossiter HB Ward SA Howe FA Kowalchuk JM Griffiths JR Whipp BJ Dynamics of intramuscular ^31^P-MRS Pi peak splitting and the slow components of PCr and O_2_ uptake during exercise. J Appl Physiol 93: 2059–2069, 2002 12391122 10.1152/japplphysiol.00446.2002

[54] Rossiter HB Ward SA Howe FA Wood DM Kowalchuk JM Griffiths JR Whipp BJ Effects of dichloroacetate on V̇o_2_ and intramuscular ^31^P metabolite kinetics during high-intensity exercise in humans. J Appl Physiol 95: 1105–1115, 2003 12754181 10.1152/japplphysiol.00964.2002

[55] Sahlin K Soderlund K Tonkonogi M Hirakoba K Phosphocreatine content in single fibers of human muscle after sustained submaximal exercise. Am J Physiol Cell Physiol 273: C172–C178, 1997 10.1152/ajpcell.1997.273.1.C172 9252454

[56] van Laar PJ van der Grond J Hendrikse J Brain perfusion territory imaging: methods and clinical applications of selective arterial spin-labeling MR imaging. Radiology 246: 354–364, 2008 18227536 10.1148/radiol.2462061775

[57] Vandenborne K Walter G Leigh JS Goelman G pH heterogeneity during exercise in localized spectra from single human muscles. Am J Physiol Cell Physiol 265: C1332–C1339, 1993 10.1152/ajpcell.1993.265.5.C1332 8238485

[58] Vanhatalo A Fulford J DiMenna FJ Jones AM Influence of hyperoxia on muscle metabolic responses and the power-duration relationship during severe-intensity exercise in humans: a 31P magnetic resonance spectroscopy study. Exp Physiol 95: 528–540, 2010 20028850 10.1113/expphysiol.2009.050500

[59] Westerblad H Allen DG Lannergren J Muscle fatigue: lactic acid or inorganic phosphate the major cause? News Physiol Sci 17: 17–21, 2002 11821531 10.1152/physiologyonline.2002.17.1.17

[60] Whipp BJ Davis JA Torres F Wasserman K A test to determine parameters of aerobic function during exercise. J Appl Physiol 50: 217–221, 1981 6782055 10.1152/jappl.1981.50.1.217

[61] Whipp BJ Rossiter HB Ward SA Exertional oxygen uptake kinetics: a stamen of stamina? Biochem Soc Trans 30: 237–247, 2002 12023858 10.1042/

[62] Whipp BJ Rossiter HB Ward SA Avery D Doyle VL Howe FA Griffiths JR Simultaneous determination of muscle ^31^P and O_2_ uptake kinetics during whole body NMR spectroscopy. J Appl Physiol 86: 742–747, 1999 9931216 10.1152/jappl.1999.86.2.742

[63] Wilson JR McCully KK Mancini DM Boden B Chance B Relationship of muscular fatigue to pH and diprotonated Pi in humans: a ^31^P-NMR study. J Appl Physiol 64: 2333–2339, 1988 3403417 10.1152/jappl.1988.64.6.2333

[64] Wray DW Nishiyama SK Monnet A Wary C Duteil S Carlier PG Richardson RS Multiparametric NMR-based assessment of skeletal muscle perfusion and metabolism during exercise in elderly persons: preliminary findings. J Gerontol A Biol Sci Med Sci 64: 968–974, 2009 19377015 10.1093/gerona/glp044 PMC2720884

[65] Wray DW Nishiyama SK Monnet A Wary C Duteil SS Carlier PG Richardson RS Antioxidants and aging: NMR-based evidence of improved skeletal muscle perfusion and energetics. Am J Physiol Heart Circ Physiol 297: H1870–H1875, 2009 19767527 10.1152/ajpheart.00709.2009 PMC2781367

[66] Yamashita H Sata M Sugiura S Momomura S Serizawa T Iizuka M ADP inhibits the sliding velocity of fluorescent actin filaments on cardiac and skeletal myosins. Circ Res 74: 1027–1033, 1994 8187272 10.1161/01.res.74.6.1027

